# Altered Spontaneous Brain Activity in Left-Behind Children: A Resting-State Functional MRI Study

**DOI:** 10.3389/fneur.2022.834458

**Published:** 2022-03-29

**Authors:** Yu Wang, Yi Lu, Meimei Du, Nimo Mohamed Hussein, Lan Li, Yu Wang, Chuanwan Mao, Tao Chen, Fangfang Chen, Xiaozheng Liu, Zhihan Yan, Yuchuan Fu

**Affiliations:** ^1^Department of Radiology, The Second Affiliated Hospital and Yuying Children's Hospital of Wenzhou Medical University, Wenzhou, China; ^2^Department of Radiology, The First Affiliated Hospital, Zhejiang University School of Medicine, Hangzhou, China; ^3^China-USA Neuroimaging Research Institute, The Second Affiliated Hospital and Yuying Children's Hospital of Wenzhou Medical University, Wenzhou, China

**Keywords:** resting-state, functional magnetic resonance imaging, amplitude of low-frequency fluctuation, fractional amplitude of low-frequency fluctuation, left behind children

## Abstract

**Background:**

Parental migration has been associated with a higher risk of cognitive and behavioral abnormalities in left-behind children (LBC). This study aimed to explore the spontaneous brain activity in LBC and reveal the mechanisms underlying behavioral and cognitive abnormalities.

**Methods:**

Involved LBC (*n* = 36) and non-LBC (*n* = 22) underwent resting-state functional MRI (fMRI) examination and cognitive and behavioral assessment. The fMRI-based amplitude of low-frequency fluctuation (ALFF) and fractional ALFF (fALFF) were assessed to analyze the spontaneous brain activity pattern. The relationships among abnormal spontaneous brain activity, behavioral and cognitive deficits and altered family environment were assessed by partial correlation analysis.

**Results:**

Compared with non-LBC, LBC exhibited increased amplitude of low-frequency fluctuations in the right lingual gyrus (LING), while a decreased ALFF was observed in the bilateral insula and right orbital part of the middle frontal gyrus (ORBmid) (two-tailed voxel-level *p* < 0.01 and cluster-level *p* <0.05, Gaussian Random Field (GRF) correction). The fALFF in LBC were significantly increased in the left cerebellum 9 (Cbe9) and right cerebellum Crus2 (CbeCru2), while it decreased in the right hippocampus and left superior temporal gyrus (STG) (two-tailed voxel-level *p* < 0.01 and cluster-level *p* < 0.05, GRF correction). The ALFF and fALFF values in abnormal brain regions were found to be correlated with the learning ability, except for the right insula, while the fALFF values of the left STG were positively correlated with the full-scale IQ scores (*p* < 0.05). Moreover, the ALFF and fALFF values in all abnormal brain regions correlated with the education level of caregivers (*p* < 0.05).

**Conclusions:**

Our study provided empirical evidence that the lack of direct parental care during early childhood could affect brain function development involving cognition, behavior, and emotion. Our findings emphasized that intellectual and emotional cares are essential for LBC.

## Introduction

In recent years, the wave of migration for better job opportunities and higher income globally has led to many children being raised by their relatives, mostly grandparents (1). Children with one or both parents moving away from them for at least 6 months are defined as left-behind children (LBC) (1). An increasing body of evidence suggests that LBC have a relatively poor quality of life (2, 3) and are prone to mental and behavioral problems, such as anxiety, depression, suicidal ideation, and conduct disorders (4, 5). These children also exhibit worse social skills and academic performance (1, 3). Furthermore, it has been established that the left-behind experience during childhood has a long-term negative impact on adolescence and adulthood (1).

Interestingly, animal studies have shown that deprivation of parental care can affect the brain development of the offspring, characterized by the death of neurons in the cortex and white matter tracts (6), the reduction of volume of the hippocampus (7), and decreased cortical thickness (8), consistent with findings in human studies. Children with institutional upbringing experience exhibit a decrease in total intracranial volume (9) and cortical thickness (10), as well as damaged integrity of white matter fiber tracts (11–13). In addition, functional MRI (fMRI) studies documented accelerated maturation of amygdala–prefrontal connectivity (14) and abnormal activation of the brain regions, such as the hippocampus, prefrontal cortex, and insula (15, 16) in children and adolescents who are subjected to threatening or cognitive tasks.

However, the above-mentioned findings were based on children raised by social institutions that cannot fully reflect the characteristics of brain structure and function of LBC. Indeed, children who grow up in social institutions face a worse living environment and lack personalized care and attention than LBC who are raised by grandparents or other relatives. In this regard, the living environment has been reported to influence the early development of the brain (17).

To the best of our knowledge, few studies have focused on the brain structure and function of LBC to assess the effect of intergenerational upbringing. In a previous study, we documented brain changes between LBC raised by grandparents and non-LBC. The structural MRI found that the brain structure of LBC was abnormal, with a larger gray matter volume in the cortical-striatal-thalamic-cortical and emotional circuit, as well as an altered fractional anisotropy in white matter tracts (18). Interestingly, after an analytical method based on graph theory was applied to resting-state fMRI data to study the topological properties of the brain, it was found that the brain function network of LBC was impaired, with an increased normalized characteristic path length and altered nodal centralities in the fronto-limbic regions and motor and sensory systems (19). However, little is currently known on the relationship between spontaneous brain activity in LBC, warranting further studies.

The amplitude of low-frequency fluctuations (ALFF) and fractional ALFF (fALFF) based on resting-state fMRI represent a reliable approach to monitor the spontaneous fluctuation of neurons and reflect the brain's physiological state (20, 21). This approach has been widely used to study brain spontaneous activity in children with behavioral and cognitive abnormality, such as drug abuse disorders (22), depression (23), impulsive behaviors (24), etc. This study aimed to explore changes in the local spontaneous brain activity of LBC *via* the ALFF/fALFF method and their association with behavior, cognition, and living environment. Given that we previously established that the brain structure and function in subjects without parental care was altered, we hypothesized that these subjects possess abnormal spontaneous activities of brain areas related to cognition, behavior, and emotion (i.e., prefrontal cortex and hippocampus). We also hypothesized that these abnormal brain areas are correlated with behavioral and cognitive assessment scores and family environment factors.

## Materials and Methods

### Participants

From January 2017 to October 2020, LBC and non-LBC (aged: 6–12 years) were recruited from the local communities in Wenzhou City, Zhejiang Province by advertising. All participants were recruited from a charity held by the Wenzhou Federation of overseas Chinese with the help of a pediatrician experienced in assessing pediatric behavioral and psychological development. All participants were from the same school. Inclusion criteria for LBC were children living apart from one or both parents for at least 6 months and raised by grandparents. The criteria for non-LBC were children raised by their parents. The exclusion criteria consisted of participants with (1) psychiatric or neurologic illness, (2) abnormalities of brain structure, such as tumors, trauma, and infections, (3) other diseases that may affect brain activity (e.g., precocious puberty and idiopathic short stature), (4) drug or alcohol addictions, and/or (5) intolerance to MRI examination. Each participant underwent MRI scans and completed behavioral and cognitive assessments and Hamilton Depression/Anxiety Scale (HAMD/HAMA) within a week. We also collected family environment factors, including education level of caregivers, family income, separation time, and age at parental departure. This work was based on the institutional guidelines of the research ethics committee of Second Affiliated Hospital and Yuying Children's Hospital of Wenzhou Medical University. Written informed consent was obtained from each participant's parent or legal guardian.

### Behavioral and Cognitive Assessment

The behavioral assessment was conducted with the Child Behavior Checklist (CBCL) in terms of activities, social contact, and learning ability (25). The Strengths and Difficulties Questionnaire (SDQ), which is a self-reporting behavioral screening questionnaire, was used to evaluate the children's attentional and behavioral problems (26). The intelligence quotient (IQ) was assessed using the 4^th^ edition of Wechsler Intelligence Scales for Children (WISC-IV, Wechsler, 2003). The assessing results included Full-Scale IQ, Verbal Comprehension Index, Perceptual Reasoning Index, Working Memory Index, and Processing Speed (27).

### MRI Acquisition

Magnetic resonance imaging was performed on a 3 Tesla scanner (Discovery 750, GE Healthcare, USA) with a 32-channel phased-array head coil. Participants were instructed to lie still, close their eyes, and keep calm. Noise reduction earplugs were used to protect the auditory system and foam padding to reduce head movement. Before the fMRI examination, routine head MRI images including T1, T2, T2 Flair, and diffusion-weighted images were acquired and examined by an experienced radiologist to ensure each enrolled participant had no structural brain abnormalities. High-resolution volumetric 3D T1-weighted imaging data were acquired by a spoiled gradient recall (SPGR) sequence in the sagittal orientation and the acquisition parameters were as follows: slices = 188, field of view (FOV) = 256 × 256 mm, echo time (TE) = 3.4 ms, repetition time (TR) = 7.7 ms, flip angle (FA) = 9°, matrix size = 256 × 256, slice thickness = 1 mm, and having no gap. The resting-state fMRI images were acquired axially for the whole brain with a gradient echo-planar imaging sequence using the following parameters: slices = 54, FOV = 216 × 216 mm, TE = 30 ms, TR = 2,500 ms, flip angle (FA) = 90°, matrix size = 72 × 72, slice thickness = 3 mm, and having no gap.

### MRI Data Preprocessing

The resting-state fMRI data were preprocessed using Data Processing Assistant for Resting-State fMRI Advanced (DPARSFA version 4) (http://www.restfmri.net) and Statistical Parametric Mapping (SPM version 8) (http://www.fil.ion.ucl.ac.uk/spm). The first ten time points were discarded to minimize the interference caused by the surrounding environment to the subjects and obtain a stable magnetization level. The remaining volumes underwent slice timing and head motion correction. The head movement was <3 mm translation or 3° angular rotation in three directions or axes. Then, they were normalized in the standard Montreal Neurological Institute (MNI) space with a resampling resolution of 3 × 3 × 3 mm and spatially smoothed with a 6-mm full width at half maximum (FWHM) kernel. Finally, linear detrending and bandpass filtering (0.01 to 0.08 Hz) were used for the data.

### ALFF and fALFF Analysis

Both ALFF and fALFF were analyzed using DPARSFA software. To obtain a power spectrum, the time series of preprocessed data were converted to the frequency domain by fast Fourier transform (FFT) (28). The square root of each frequency of the power spectrum was calculated, and the average square root of the spectrum ranging from 0.01 to 0.08 Hz of each voxel was analyzed as ALFF (28). The fALFF was obtained by dividing ALFF by the sum of the amplitudes of the whole frequency band to effectively reduce the influence of respiratory and cardiac signals (29).

### Statistical Analysis

For statistical analysis, two-sample *t*-tests were performed by Resting-State fMRI Data Analysis Toolkit (REST version 1.8) (http://www.restfmri.net/) to determine the significance of differences in ALFF and fALFF between the LBC and non-LBC groups with sex and age as nuisance covariates. Multiple comparisons correction using Gaussian Random Field (GRF) correction (two-tailed voxel-level *p* < 0.01 and cluster-level *p* < 0.05) was conducted by REST software (30). A Kolmogorov-Smirnov test was performed to assess the normality of clinical variables. Then, clinical variables were analyzed by two-sample *t*-tests or Mann-Whitney U tests to compare the differences between the two groups. The analysis of gender differences was based on a chi-square test. Furthermore, a partial correlation analysis (adjusting for sex and age) was performed to assess the associations between altered spontaneous brain activity (ALFF and fALFF values), behavioral and cognitive scores, and significant family environment factors between the two groups. Furthermore, mediation analysis was conducted to assess the relationship between an outcome variable (behavioral and cognitive level) and independent variables (spontaneous brain activity) *via* a mediator (environment factors), after adjusting for age and gender. All statistical analyses were performed with SPSS version 22 (Chicago, IL, USA), and a *p*-value < 0.05 was statistically significant.

## Results

### Group Characteristics

Left-behind children raised by grandparents (*n* = 36, 7.27–11.92 years old; mean ± *SD* = 9.19 ± 1.31) and non-LBC (*n* = 22, 7.17–11.25 years old; mean ± *SD* = 9.59 ± 1.02) were included in this study. All children were right-handed and studied in primary school at enrollment. There were significant differences in the education level of caregivers and family income between LBC and non-LBC groups (*p* < 0.05). No difference in age, sex, weight, height, body mass index (BMI), birth weight, and the type of delivery between the two groups (*p* > 0.05). The average age of the LBC group at parental departure was 17.89 months, while the mean separation time was 7.4 years. The group characteristics are displayed in Table 1.

**Table 1:** Demographic information of left-behind children (LBC) and healthy controls (HC).

**Variables (mean ±SD)**	**LBC**	**non-LBC**	***p*** **-value**
Age (years)	9.19 ± 1.31	9.59 ± 1.02	0.23
Sex (male/female)	18/18	10/12	0.74
Weight (kg)	31.00 ± 7.86	31.02 ± 7.33	0.94
Height (m)	1.38 ± 0.11	1.37 ± 0.06	0.67
BMI (kg/m^2^)	15.98 ± 2.36	16.25 ± 2.29	0.58
Birth weight (kg)	3.20 ± 0.37	3.35 ± 0.42	0.10
Delivery (labor/cesarean)	32/4	18/4	0.48
Education of caregivers (years)	2.46 ± 2.17	15.5 ± 2.79	0.00*
Family income (10,000 yuan/year)	20.28 ± 8.19	31.52 ± 16.46	0.00*
Separation time (years)	7.40 ± 2.25		
Age at parental departure (months)	17.89 ± 19.56		

Values are mean ± SD. BMI, Body Mass Index. The symbol ^*^ denotes p < 0.05.

### Differences in Behavioral and Cognitive Levels

Left-behind children exhibited significantly lower activity and learning ability scores than non-LBC during CBCL assessment (*p* < 0.05). Scores for full-scale IQ, Verbal Comprehension Index, Perceptual Reasoning Index, and Working Memory Index of WISC-IV were also lower in LBC (*p* < 0.05). No significant difference in social skills and total CBCL were found during CBCL assessment. Moreover, the Processing Speed Index used in WISC-IV and the SDQ subscale scores were comparable between LBC and non-LBC groups (*p* > 0.05). Table 2 provides information on the behavioral and cognitive assessment between the two groups.

**Table 2:** Behavioral and cognitive assessment of LBC and HC.

**Variables (mean ±SD)**	**LBC**	**non-LBC**	***p*** **-value**
**CBCL**			
Activity	3.25 ± 2.50	6.16 ± 2.40	0.00*
Social skills	6.31 ± 2.17	7.06 ± 2.48	0.23
Learning ability	4.89 ± 0.69	5.77 ± 0.42	0.00*
Total score	8.86 ± 7.92	9.91 ± 7.92	0.57
**WISC-IV**			
Verbal Comprehension	98.06 ± 12.18	111.68 ± 18.66	0.00*
Perceptual Reasoning	102.19 ± 10.35	111.55 ± 12.72	0.00*
Working Memory	94.89 ± 9.52	103.59 ± 20.73	0.01*
Processing Speed	100.25 ± 10.19	103.82 ± 19.59	0.37
Full-Scale IQ	98.56 ± 10.00	112.91 ± 15.52	0.00*

Values are mean ± SD. CBCL, Child Behavior Checklist; WISC-IV, Wechsler Intelligence Scale for Children, fourth edition. The symbol ^*^ denotes p < 0.05.

### Abnormal Spontaneous Brain Activity

**Figure 1:**
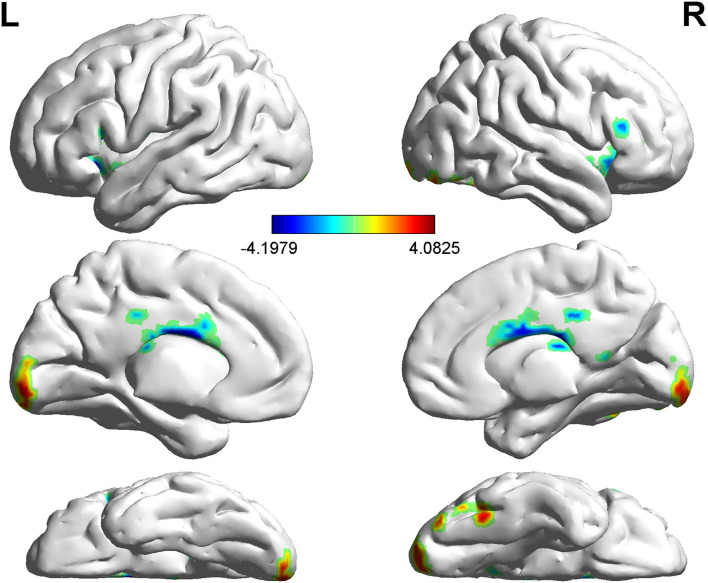
Comparison of amplitude of low-frequency fluctuation (ALFF) between two groups. The figure showed increased ALFF in left-behind children (LBC) in the right LING and decreased ALFF in the bilateral insula and right ORBmid (two-tailed voxel-level *p* < *0.0*1 and cluster-level *p* < 0.05, GRF correction). LING, lingual gyrus; ORBmid: orbital part of middle frontal gyrus; GRF, Gaussian Random Field.

**Figure 2:**
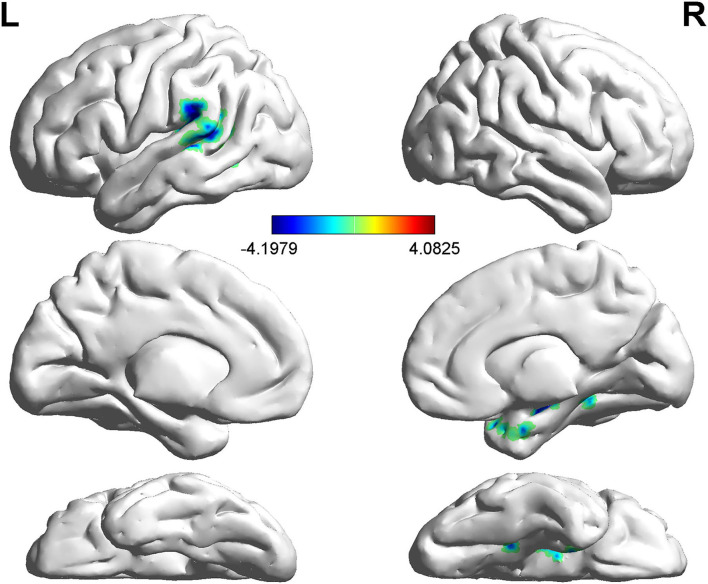
Comparison of fractional ALFF (fALFF) between two groups. The figure showed decreased fALFF in the right hippocampus and left STG (two-tailed voxel-level *p* < 0.01 and cluster-level *p* < 0.05, GRF correction). Cbe9, cerebellum 9; CbeCru2, cerebellum crus2; STG: superior temporal gyrus; GRF: Gaussian Random Field.

Compared with non-LBC, LBC exhibited increased amplitude of low-frequency fluctuations in the right lingual gyrus (LING), while a decreased ALFF was observed in the bilateral insula and right orbital part of the middle frontal gyrus (ORBmid) (two-tailed voxel-level *p* < 0.01 and cluster-level *p* < 0.05, GRF correction) (Table 3, Figure 1). In addition, compared with non-LBC, the fALFF in LBC was significantly increased in regions located in the left cerebellum 9 (Cbe9) and right cerebellum crus2 (CbeCru2) and decreased in the right hippocampus and left superior temporal gyrus (STG) (two-tailed voxel-level *p* < 0.01 and cluster-level *p* < 005, GRF correction) (Table 4, Figure 2).

**Table 3:** Brain regions showing significant differences in amplitude of low-frequency fluctuation (ALFF) between LBC and non-LBC.

			**MNI coordinates**	
**Regions**	**BA**	**Voxels**	**(x,y,z)**	**Peak *t*-value**
R-LING	18	89	15,-99,-12	4.0825
R-INS	47	49	30,6,-9	−3.3716
R-ORBmid	11	57	15,39,-9	−4.1606
L-INS	47	50	−30,21,-6	−3.7633
L-INS	48	40	−39,18,9	−3.2982

*A significance level of two-tailed voxel-level p < 0.01 and cluster-level p < 0.05, was set for GRF correction. R, right; L, left; LING, lingual gyrus; INS, insula; ORBmid, orbital part of middle frontal gyrus; GRF, Gaussian Random Field*.

**Table 4:** Brain regions showing significant differences in fALFF between LBC and non-LBC.

			**MNI coordinates**	
**Regions**	**BA**	**Voxels**	**(x,y,z)**	**Peak *t*-value**
L-Cbe9		38	3,-54,-60	3.2218
R-CbeCru2		139	0,-81,-36	3.7732
R-hippocampus	20	94	36,-18,-9	−4.1908
L-STG	41	126	−45,-33,15	−3.9850

*A significance level of two-tailed voxel-level p < 0.01 and cluster-level p < 0.05, was set for GRF correction. R, right; L, left; Cbe9, cerebellum 9; CbeCru2, cerebellum Crus2; STG, superior temporal gyrus; GRF, Gaussian Random Field*.

### Correlations Between ALFF and fALFF Values and Different Behavioral and Cognitive Levels and Family Environment

The ALFF and fALFF values in abnormal brain regions were found to be correlated with the learning ability during CBCL assessment, except for the right insula. Moreover, the fALFF values in the left STG positively correlated with the full-scale IQ scores WISC-IV (*p* < 0.05) (Table 5). Furthermore, all altered ALFF and fALFF values correlated with the education level of caregivers (*p* < 0.05) (Table 5). The correlations between learning ability or full-scale IQ and spontaneous brain activity changes (except for the ALFF changes in the right ORBmid) could be explained to a certain extent by the education level of caregivers (Table 6).

**Table 5:** Association between values of ALFF and fALFF and living environment and behavioral and cognitive assessment.

	**Life environment**	**CBCL**	**WISC-IV**
	**Education of caregivers**	**Learning ability**	**Full-Scale IQ**
	**r *p***	**r *p***	**r *p***
**ALFF**			
R-LING	−0.432 0.001	−0.297 0.026	
R- insula	0.415 0.001		
R-ORBmid	0.317 0.017	0.428 0.001	
L- insula	0.428 0.001	0.346 0.009	
L- insula	0.387 0.003	0.376 0.004	
**fALFF**			
L-Cbe9	−0.377 0.004	−0.275 0.040	
R-CbeCru2	−0.467 0.000	−0.331 0.013	
R-hippocampus	0.419 0.001	0.291 0.030	
L-STG	0.400 0.002	0.376 0.004	0.326 0.014

*CBCL, Child Behavior Checklist; WISC-IV, Wechsler Intelligence Scale for Children, fourth edition; R, right; L, left; LING, lingual gyrus; ORBmid, orbital part of middle frontal gyrus; Cbe9, cerebellum 9; CbeCru2, cerebellum Crus2; STG, superior temporal gyrus*.

**Table 6:** Mediation effects of environmental factors on the correlation between spontaneous brain activity and behavioral and cognitive level.

	**Total effect**	**Indirect effect**	**Direct effect**	**Percent mediation (%)**
**ALFF**				
R-LING—Learning ability	−0.4493* (-0.8429,−0.0557)	−0.3097 (-0.6056,−0.1244)	−0.1397 (-0.5336, 0.2543)	68.93
R-ORBmid—Learning ability	1.6425** (0.6950, 2.5900)	0.5124 (0.0511, 1.0776)	1.1301 (0.2252, 2.0350)	31.20
L-insula—Learning ability	1.0427* (0.2709, 1.8145)	0.5784 (0.2532, 1.1122)	0.4643 (-0.3134, 1.2420)	55.13
**fALFF**				
L-Cbe9—Learning ability	−0.7933* (-1.5493,−0.0372)	−0.5201 (-1.0558,−0.1467)	−0.2732 (-1.0047, 0.4583)	65.56
R-CbeCrus2—Learning ability	−0.9239* (-1.6421,−0.2056)	−0.5992 (-1.0442,−0.2690)	−0.3247 (-1.0648, 0.4154)	64.86
R-hippocampus—Learning ability	2.3111* (0.2376, 4.3846)	1.5859 (0.7614, 3.0494)	0.7252 (-1.3326, 2.7831)	68.62
L-STG—Learning ability	2.6695** (0.8742, 4.4647)	1.2315 (0.4485, 2.3533)	1.4380 (-0.3496, 3.2255)	46.13
L-STG—Full-Scale IQ	43.8218* (9.2070, 78.4367)	23.3899 (6.7706, 47.1709)	20.4319 (-14.1499, 55.0137)	53.38

Effects represent as β (95% CI). The symbols * and ** denote p < 0.05 and < 0.01, respectively. R, right; L, left; LING, lingual gyrus; ORBmid, orbital part of middle frontal gyrus; Cbe9, cerebellum 9; CbeCru2, cerebellum Crus2; STG, superior temporal gyrus.

## Discussion

Our current study used the ALFF/fALFF method to analyze the spontaneous activity in the brain of LBC. We found abnormal spontaneous activity in some brain regions related to cognition and emotion in LBC, including prefrontal-limbic regions (ORBmid, hippocampus, and insula), LING, and CbeCru2, among others. Furthermore, changes in spontaneous brain activity were correlated with the learning ability, full-scale IQ scores, and education level of caregivers.

We found that brain regions exhibiting abnormal spontaneous activity in LBC belonged to the prefrontal-limbic system with decreased ALFF on the bilateral insula and right ORBmid, as well as on the fALFF of the right hippocampus. Previous studies reported that early deprivation of parental care could induce changes in the hippocampus and prefrontal cortex in the offspring. In this respect, in rats with a 24 h maternal deprivation, the thickness and cell soma area of the prefrontal cortex and the volume of the hippocampus were decreased (7, 8). Consistently, studies on institutionalized children found reductions in prefrontal cortex thickness (10) and hippocampal volume (9). Rao et al. (31) also found that the hippocampal volume was specifically associated with early parental nurturance. It is highly conceivable that changes in the spontaneous activity of the prefrontal cortex and hippocampus might be related to their structural destruction.

The study of Zhao et al. (19) also confirmed the presence of disordered nodal centralities of fronto-limbic regions in LBC. It has been shown that the hippocampus and insula participate in cognition with memory function and play a large role in executing emotion (32, 33). Moreover, the orbitofrontal cortex is considered anatomically suitable for integrating cognitive and emotional functions (33). In adolescents with a parent deprivation experience, the increased activation of the left anterior hippocampus was documented when processing threatening messages (15). There is also an abnormal activation in the inferior prefrontal cortex and the posterior insula of these adolescents when facing cognitive tasks (16).

Our study found that compared with non-LBC, changes in the spontaneous activity of LING, STG, left Cbe9, and right CbeCru2 were observed in LBC. Notwithstanding that they all participate in cognition, these brain parts play different roles associated with cognitive function. LING has been reported to play an important role during early antidepressant treatment and maintenance of cognitive function (34). The study conducted by Zhao et al. (19) found that the nodal degree, efficiency, and betweenness of the LING were increased in LBC compared with non-LBC. STG has been supposed to participate in social cognition (35, 36). It has been reported that there is a long loop regulation that returns from the cerebellum (i.e., the cognitive-related areas of CbeCru2) to the cerebral cortex in human cognition (37).

Behavioral and cognitive problems in LBC were found in this study, characterized by lower learning ability and full-scale IQ scores. Moreover, the learning ability and full-scale IQ were related to abnormal spontaneous activity in the brain regions. Furthermore, our study found that the education level of caregivers correlated with abnormal spontaneous activity in brain regions and accounted for the correlations between learning ability or full-scale IQ and spontaneous brain activity changes to a certain extent. The educational level of the primary caregiver in the LBC group was limited to the primary school, and some primary caregivers were even illiterates. Consistently, it has been reported that guardians with lower educational levels are more likely to adopt inappropriate educational methods, such as beating and scolding, which may easily bring pressure to children and affect their brain development (38).

There were several limitations in our study. First of all, given the cross-sectional observational nature of our study, we could not explore whether the impact of the left-behind experience on the brain could last into adolescence or even adulthood, emphasizing the need for a longitudinal study. Moreover, the sample size was relatively small. Larger sample size will be analyzed in our future studies. Finally, the behavioral and cognitive assessment was subjective. Accordingly, the same experienced pediatrician evaluated and provided appropriate guidance to all participants and their guardians.

## Conclusion

Left-behind children are more likely to have cognitive, behavioral, and psychological problems than non-LBC. Our study provided empirical evidence that the lack of direct parental care during early childhood could affect brain function development involving cognition, behavior, and emotion. Importantly, programs providing more intellectual and emotional care are needed for LBC to reduce the potential long-term adverse consequences on this particular population.

## Data Availability Statement

The datasets generated for this study are available on request to the corresponding authors.

## Ethics Statement

The studies involving human participants were reviewed and approved by the Research Ethics Committee of Second Affiliated Hospital and Yuying Children's Hospital of Wenzhou Medical University. Written informed consent to participate in this study was provided by the participants' legal guardian/next of kin.

## Author Contributions

YF and ZY: experimental design. YW (1st author), YL, NH, MD, and CM: MRI data collection. LL and YW (6th author): clinical data collection. TC and FC: data curation. XL and YW (1st author): data analysis. YW (1st author) and YL: manuscript preparation. All authors contributed to and have approved the final manuscript.

## Funding

This work was supported by the National Natural Science Foundation of China (Grant No. 82071902), the Natural Science Foundation of Zhejiang Province (Grant No. LY19H180003), and the Medical Health Science and Technology Project of Zhejiang Province (Grant Nos. 2017KY108, 2017ZD024, and 2015KYB250).

## Conflict of Interest

The authors declare that the research was conducted in the absence of any commercial or financial relationships that could be construed as a potential conflict of interest.

## Publisher's Note

All claims expressed in this article are solely those of the authors and do not necessarily represent those of their affiliated organizations, or those of the publisher, the editors and the reviewers. Any product that may be evaluated in this article, or claim that may be made by its manufacturer, is not guaranteed or endorsed by the publisher.
